# Innovative Approaches to Infectious Disease Prevention in Women^1^

**DOI:** 10.3201/eid1011.040622_06

**Published:** 2004-11

**Authors:** Marian McDonald, Joyce Hunter, Dazon Dixon Diallo, Monique Petrofsky, Hilda Seda

**Affiliations:** * Centers for Disease Control and Prevention, Atlanta, Georgia, USA;; † New York State Psychiatric Institute, New York City, New York, USA;; ‡ SisterLove, Inc., Atlanta, Georgia, USA

**Keywords:** infectious diseases, women, conference summary

Preventing infectious diseases among women requires innovative and empowering community-based strategies. Strategies that involve women in the design, staffing, implementation, and evaluation of programs can result in greater effectiveness and empowerment and reduction of infectious disease transmission.

## Community Capacity Building

Community capacity building is the process of enhancing a community's ability to respond to challenges and opportunities and to address its needs through the development of leaders, skills, knowledge, and resources. It is essential to community-based infectious disease prevention. SisterLove is a women's organization dedicated to eradicating the effects of HIV/AIDS and other reproductive health challenges on women and their families through education, prevention, support, and human rights advocacy in the United States and around the world. The work at SisterLove focuses on women and HIV through health education, advocacy, housing, and support services; the work is performed through grassroots, community-based organizations. SisterLove is involved in international programs, such as the Thembuhlelo HIV/AIDS Capacity Building Project in South Africa, and bridge leadership, such as the Women's HIV/AIDS Resources Project. SisterLove uses grassroots, community-based organizations as primary agents because these places are where people seek services. Three key elements to the success of this program are the following: the programs must be inclusive of the communities in which they are working, staff members must relate to the people in the communities, and staff members must not set up false expectations and must deliver on promises.

## Guinea Worm Eradication Program

The Guinea worm eradication program in Ghana has partnered with the Ghana Red Cross women's clubs to reduce transmission of Guinea worm. The partnership involved teaching village-based women volunteers how Guinea worm is transmitted and how to prevent transmission. Red Cross women volunteers educate communities on methods to prevent transmission.

Traditionally, Guinea worm eradication efforts worked primarily with male volunteers, which presented a variety of challenges because men are frequently required to travel outside the village and are not responsible for gathering water. Since women are primarily responsible for water gathering and using the water for household consumption, providing ownership in the program was important for the success of the project. By engaging women volunteers, village women were convinced to use the filters and to not contaminate the water.

Responsibilities of the women volunteers include daily surveillance by finding, identifying, and reporting all new cases; case containment by ensuring that infected persons do not contaminate water sources; and distributing, checking, and replacing specifically manufactured filters that filter out the copepods. Women volunteers also assisted in identifying water resources used by the community for larvicidal treatment. Through this program, cases of Guinea worm have decreased dramatically.

## Role of Women in Dengue Prevention

Studies in Puerto Rico and elsewhere in Latin America have documented the role of women in preventing dengue and dengue hemorrhagic fever. When properly educated and motivated, many women have contributed to the development and promotion of educational interventions that are used to control or eliminate the mosquito vector of dengue. After receiving training to develop communication, education, and community organizational skills, a group of mothers of children enrolled in the Head Start program conducted four dengue prevention activities in their community. Their intervention resulted in higher levels of knowledge about dengue when compared to a control community. This study demonstrated that a small group of motivated women can generate a good response in their community. For greater effect and to increase the number of people exposed to educational messages, more long-term prevention activities need to be promoted in the community.

